# Echocardiographic Left Ventricular Hypertrophy Among Patients with Type 2 Diabetes Undergoing Clinically Indicated Echocardiography at a Saudi Cardiac Center: Prevalence and Associated Factors

**DOI:** 10.3390/biomedicines14102308

**Published:** 2026-10-11

**Authors:** Jolan Alsaud, Mohammed Aldkheel, Fatimah Alenazi, Razan Alharbi, Reef Alsuhaibani, Joud Almotairi, Sali Alharbi, Lyan Alharbi, Emtenan Alshalahi, Alwaleed Alamro, Metab Algeffari

**Affiliations:** 1 Family Medicine, Qassim Health Cluster, Buraydah 52387, Saudi Arabia; 2 Prince Sultan Cardiac Center, Qassim Health Cluster, Buraydah 52387, Saudi Arabia; 3 Department of Public Health, College of Applied Medical Sciences, Qassim University, Buraydah 52387, Saudi Arabia; 4 Department of Family and Community Medicine, College of Medicine, Qassim University, Buraydah 52387, Saudi Arabia; 5 Abdullah Al Othaim Diabetes Center, Qassim University Medical City, Qassim University, Buraydah 52387, Saudi Arabia

**Keywords:** type 2 diabetes mellitus, left ventricular hypertrophy, left ventricular mass index, echocardiography, hypertension, blood pressure, cross-sectional studies, Saudi Arabia

## Abstract

**Background/Objectives**: Left ventricular hypertrophy (LVH) is an important cardiovascular complication, but regional data on this condition among patients with type 2 diabetes mellitus (T2DM) in Saudi Arabia remain limited. This study determined the prevalence of LVH and associated factors among adults with T2DM without documented cardiac disease undergoing clinically indicated echocardiography at a specialized cardiac center in Saudi Arabia. **Methods**: This retrospective cross-sectional study included 625 patients with T2DM who underwent clinically indicated echocardiography at Prince Sultan Cardiac Center, Qassim, between January 2023 and December 2025. Of 878 patients with T2DM and no documented cardiac disease assessed for eligibility, 253 (28.8%) were excluded because at least one required clinical variable was missing or unavailable, leaving 625 patients (71.2%) in the final analysis. LVH was assessed according to American Society of Echocardiography/European Association of Cardiovascular Imaging recommendations. Multivariable logistic regression was used to evaluate factors associated with LVH. **Results**: LVH was identified in 146/625 patients (23.4%; 95% CI, 20.0–26.7). After multivariable adjustment, male sex (aOR = 1.71, 95% CI: 1.11–2.63; *p* = 0.015) and uncontrolled/elevated blood pressure (aOR = 2.18, 95% CI: 1.26–3.80; *p* = 0.006) were associated with LVH. Diabetes duration showed a significant nonlinear association with LVH (overall *p* < 0.001; *p* for nonlinearity = 0.001). **Conclusions**: LVH was common among this clinically selected group of patients with T2DM undergoing clinically indicated echocardiography. Male sex and uncontrolled/elevated blood pressure were associated with LVH after adjustment, while diabetes duration showed a nonlinear association. Although causal relationships cannot be established, the observed association between uncontrolled/elevated blood pressure and LVH highlights the importance of optimal blood pressure management as part of comprehensive cardiovascular risk reduction in patients with T2DM.

## 1. Introduction

Type 2 diabetes mellitus (T2DM) is a major global health concern and an important contributor to cardiovascular morbidity and mortality [1,2], and it also represents a substantial health burden in Saudi Arabia. A systematic review and meta-analysis of observational studies conducted between 2000 and 2020 estimated a pooled T2DM prevalence of 16.4% (95% CI: 11.6–17.5) in the Saudi general population, although substantial heterogeneity was observed across studies [1].

T2DM is associated with structural and functional cardiac abnormalities, including left ventricular hypertrophy (LVH) [2,3]. LVH is clinically important because it is associated with adverse cardiovascular outcomes, including heart failure [3]. Importantly, echocardiographically determined left ventricular mass provides prognostic information beyond traditional cardiovascular risk factors, with greater left ventricular mass independently associated with cardiovascular events, cardiovascular mortality, and all-cause mortality [4]. Furthermore, diabetes has been associated with greater left ventricular mass and wall thickness even after adjustment for BMI, arterial pressure, and other relevant covariates, suggesting adverse cardiac effects beyond those attributable to the accompanying increases in BMI and arterial pressure [5]. LVH development in patients with T2DM is multifactorial, involving hemodynamic and metabolic mechanisms [3]. Accordingly, examining demographic and clinical factors such as age, sex, diabetes duration, glycemic status, obesity, hypertension (HTN) history, and contemporaneous blood pressure (BP) may help characterize the clinical profile associated with LVH in patients with T2DM. Identifying such factors may also be clinically relevant because left ventricular mass is potentially modifiable; antihypertensive therapy has been shown to reduce left ventricular mass in patients with essential HTN [6]. Moreover, lower left ventricular mass during antihypertensive treatment has been associated with lower rates of cardiovascular events beyond the effects of BP lowering [7]. These findings support the clinical relevance of identifying factors associated with LVH, particularly those that may be modifiable.

Previous studies have reported varying estimates of LVH among patients with T2DM. Parsa et al. reported a prevalence of 20.7%, whereas Mani et al. reported 25.6% [8,9]. Other studies using echocardiographic assessment have also identified LVH and other structural cardiac abnormalities in patients with T2DM [10]. However, these estimates were derived from different clinical populations and were based on different methods and criteria for identifying LVH, limiting direct comparisons across studies.

Saudi data on LVH among patients with T2DM remain geographically limited. A recent study from the Jazan region reported LVH in 30.4% of adults with T2DM undergoing echocardiographic evaluation [11]; however, evidence from other regions, including Qassim, remains scarce. Specifically, LVH and its associated factors have not been well characterized among clinically selected patients with T2DM without previously documented cardiac disease undergoing echocardiography in the Qassim region. Characterizing this referral-based population is clinically informative because it represents patients selected for echocardiographic evaluation on clinical grounds despite having no previously documented cardiac disease, thereby providing insight into LVH and its associated factors in a clinically relevant group in whom structural cardiac abnormalities may not have been previously recognized. Therefore, this study aimed to determine the prevalence of LVH and identify its associated factors in this hospital-based, clinically selected echocardiography cohort.

## 2. Materials and Methods

### 2.1. Study Design, Setting and Population

This retrospective cross-sectional study was conducted at Prince Sultan Cardiac Center, a specialized cardiac care center in the Qassim region of Saudi Arabia, and included echocardiographic examinations performed between January 2023 and December 2025. Following ethics approval on 18 May 2026, medical records corresponding to this predefined study period were retrospectively reviewed and analyzed.

Adult patients (aged ≥ 18 years) were eligible for inclusion if they had a documented diagnosis of T2DM, had undergone echocardiographic evaluation during the study period, had no previously documented cardiac disease, and had sufficient clinical and echocardiographic data for the study variables. T2DM was ascertained from the electronic medical record using diagnostic codes together with physician documentation. Patients with type 1 diabetes mellitus or other documented forms of diabetes were not included. The presence or absence of previously documented cardiac disease was similarly determined through review of diagnostic codes and physician documentation in the medical record. Patients with documented coronary artery disease, previous myocardial infarction, heart failure, cardiomyopathy, valvular heart disease, or congenital heart disease were excluded.

All echocardiographic examinations were clinically indicated and had been requested by the treating physician as part of routine clinical care rather than performed as systematic screening for patients with diabetes. Specific indications for individual echocardiographic examinations were not consistently available in the extracted analytical dataset and therefore could not be categorized retrospectively.

**Figure 1:**
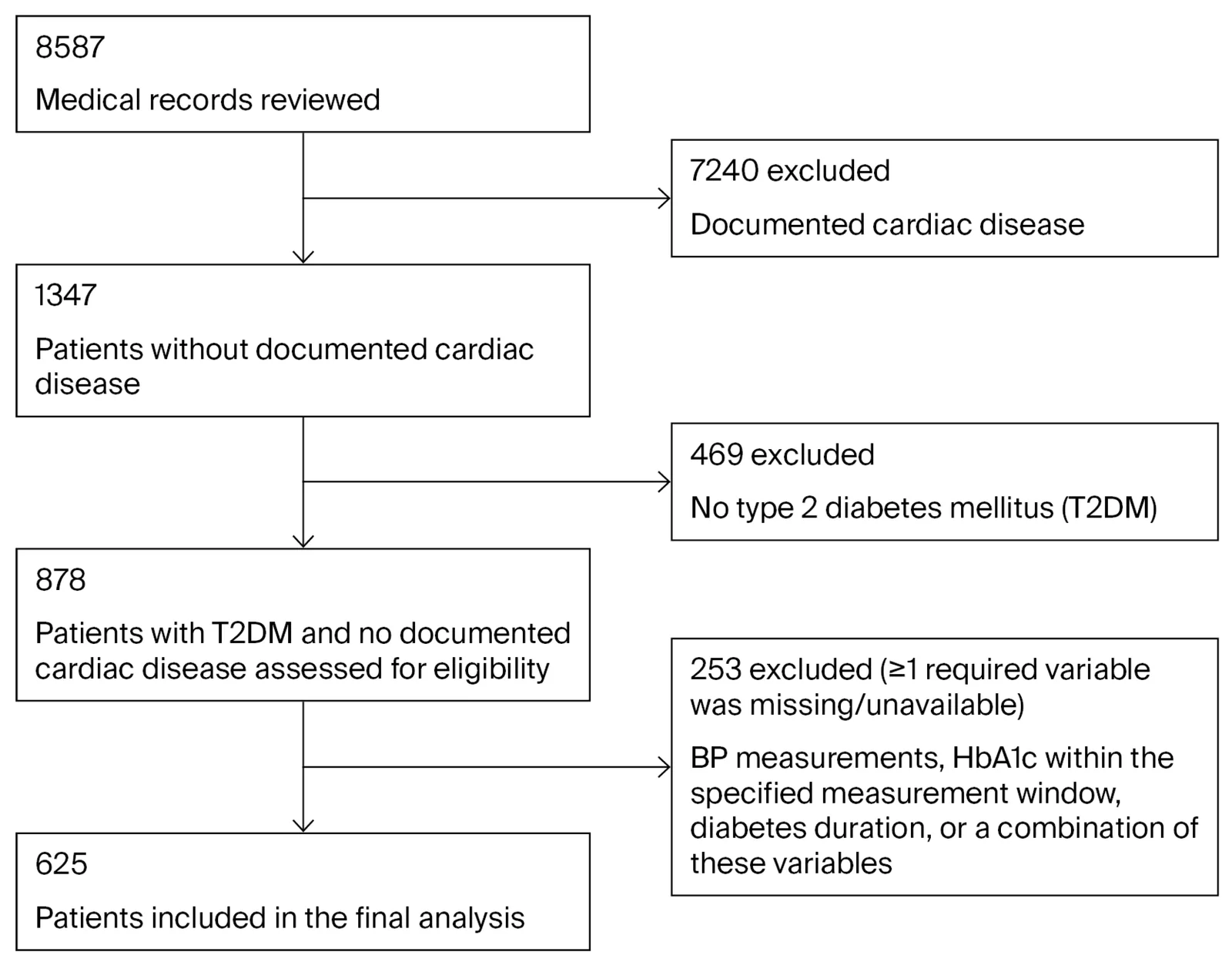
Flow diagram of study population selection.

A total of 8587 medical records were screened, of which 7240 patients with documented cardiac disease were excluded, leaving 1347 patients without documented cardiac disease. Among these, 469 patients without T2DM were excluded, leaving 878 patients with T2DM and no documented cardiac disease who were assessed for eligibility. A further 253 patients (28.8%) were excluded because at least one required variable was missing or unavailable, leaving 625 patients (71.2%) in the complete-case analysis. Missingness leading to exclusion involved one or both required BP measurements, hemoglobin A1C (HbA1c) within the specified measurement window, diabetes duration, or a combination of these variables. Because individual-level data for the excluded records were not retained in the final analytical dataset, a formal comparison between included and excluded patients and multiple imputation could not be performed. The final analytical cohort therefore comprised 625 patients (Figure 1).

A census sampling approach was used to include all eligible patients identified during the study period; therefore, no a priori sample-size calculation was performed. The observed LVH prevalence of 23.4% in the final cohort of 625 patients corresponded to a 95% CI of 20.0–26.7%, representing an achieved precision of approximately ±3.3 percentage points. With 146 LVH events and approximately nine effective predictor parameters in the final multivariable model, the analysis provided approximately 16 events per parameter.

### 2.2. Data Collection Tools and Process

Data were collected retrospectively from the patients’ medical records using a structured data collection form. The extracted demographic and clinical variables included age, sex, duration of diabetes, known history of HTN, systolic blood pressure (SBP), diastolic blood pressure (DBP), HbA1c, height, weight, BMI, and echocardiographic LVH status. Clinical variables were retrieved from measurements documented within the same calendar month as the index echocardiographic examination. Diabetes duration was extracted from the duration documented in the medical record rather than calculated from the date of diagnosis at the study center, because patients could have been diagnosed with T2DM before receiving care at our institution.

Pharmacological treatment was not consistently or completely documented in the available records and therefore could not be reliably incorporated into the analysis. Similarly, renal function, albuminuria, lipid measures, smoking status, anemia, and detailed information regarding the duration and severity of HTN were not available with sufficient completeness for inclusion in the analytical dataset.

Glycemic control was classified according to the documented HbA1c value obtained within the same calendar month as the index echocardiographic examination, with HbA1c < 7.0% considered controlled and HbA1c ≥ 7.0% considered uncontrolled [12]. A known history of HTN was recorded separately as a binary variable (yes/no).

BP was measured using a standardized clinical protocol. Before measurement, patients were instructed to avoid smoking, caffeinated beverages, alcohol, and exercise for at least 30 min and to rest quietly for more than 5 min. Measurements were obtained with the patient seated upright with the back supported, feet flat on the floor, legs uncrossed, and arm comfortably supported on a flat surface at heart level. The cuff was applied to the bare upper arm above the antecubital fossa rather than over clothing. An appropriately sized cuff was selected according to arm circumference, with bladder length and width corresponding to approximately 75–100% and 37–50% of the arm circumference, respectively. Patients were instructed to remain relaxed and not talk during measurement.

For the present study, BP status was determined from the mean of the two most recently recorded SBP and DBP measurements obtained within the same calendar month as the index echocardiographic examination. Controlled/normal BP was defined as SBP < 130 mmHg and DBP < 80 mmHg, whereas uncontrolled/elevated BP was defined as SBP ≥ 130 mmHg or DBP ≥ 80 mmHg [13]. The 130/80 mmHg threshold was selected in accordance with the guideline used for cardiovascular risk assessment in patients with diabetes [13]. History of HTN and contemporaneous BP status were retained as separate variables because they represent clinically distinct constructs: established HTN and contemporaneous BP status at the time of clinical assessment, respectively.

BMI was calculated as weight in kilograms divided by height in meters squared (kg/m^2^) and categorized as underweight (<18.5 kg/m^2^), normal weight (18.5 to <25.0 kg/m^2^), pre-obesity (25.0 to <30.0 kg/m^2^), and obesity (≥30.0 kg/m^2^) [14]. Because the underweight category contained relatively few patients, Fisher’s exact test rather than Pearson’s chi-square test was used for comparisons involving the four-category BMI variable. For multivariable regression, BMI was entered as obesity (BMI ≥ 30 kg/m^2^) versus non-obesity (BMI < 30 kg/m^2^).

Echocardiographic examinations were performed and interpreted by cardiologists at Prince Sultan Cardiac Center using the center’s standardized echocardiographic acquisition protocol and in accordance with the recommendations of the American Society of Echocardiography and the European Association of Cardiovascular Imaging (ASE/EACVI). Left ventricular mass was calculated using the ASE-recommended linear method and indexed to body surface area to derive the left ventricular mass index (LVMI). LVH was defined using sex-specific ASE/EACVI reference limits: LVMI > 115 g/m^2^ in men and >95 g/m^2^ in women [15].

For the present study, LVH status was extracted from the final cardiologist-interpreted echocardiography report and recorded as a binary variable (present or absent). Continuous LV mass and LVMI values were not retained in the analytical dataset; consequently, alternative indexation methods, such as height-based indexation, and analyses of continuous LVMI could not be performed. No study-specific re-reading or formal interobserver variability assessment was performed, because the analysis relied on the final clinically interpreted echocardiography reports.

### 2.3. Data Management and Statistical Analysis

Statistical analyses were performed using IBM SPSS Statistics version 27.0 (IBM Corp., Armonk, NY, USA). The distributions of continuous variables were assessed before analysis. Approximately normally distributed variables are summarized using means and standard deviations (SDs), whereas skewed variables are summarized using medians and interquartile ranges (IQRs), as appropriate. Categorical variables are presented as frequencies and percentages. Between-group comparisons were performed using independent-samples t-tests, with Welch’s correction when appropriate, for approximately normally distributed continuous variables and appropriate nonparametric tests for skewed continuous variables. Pearson’s chi-square test was used for categorical variables when expected cell-count assumptions were satisfied; Fisher’s exact test was used when these assumptions were not met, including comparisons involving the four-category BMI variable.

Multivariable logistic regression was used to evaluate factors associated with LVH. Prespecified covariates were entered simultaneously and included age, sex, diabetes duration, glycemic control, obesity, known history of HTN, and contemporaneous BP status. Age was modeled continuously rather than dichotomized. The linearity of continuous predictors on the logit scale was assessed before final model specification. Age showed no evidence of departure from linearity, whereas diabetes duration demonstrated a nonlinear association with LVH and was therefore modeled using a natural cubic spline with 4 degrees of freedom rather than assuming a constant linear effect. The overall association and test for nonlinearity were reported for diabetes duration. For graphical presentation, adjusted odds ratios and 95% confidence intervals across the observed range of diabetes duration were estimated from the fitted spline model, using 10 years of diabetes duration as the reference value.

Known history of HTN and contemporaneous BP status were included simultaneously because they represent distinct clinical constructs: established HTN and contemporaneous BP status at the time of clinical assessment, respectively. A sensitivity model excluding HTN history was fitted to assess the robustness of the association between current BP status and LVH. Additional sensitivity analyses replaced the binary BP-status variable with continuous SBP, DBP, and pulse pressure in separate models; SBP and DBP were also evaluated jointly in a supplementary model.

Prespecified interactions between sex and BP status and between diabetes duration and glycemic control were evaluated. Multicollinearity was assessed using variance inflation factors (VIFs). Model discrimination was assessed using the C-statistic, and calibration was evaluated using the Hosmer–Lemeshow goodness-of-fit test. Adjusted odds ratios (aORs) with 95% CIs were reported for categorical and linearly modeled predictors; for diabetes duration, the overall association and nonlinearity test were reported because it was modeled nonlinearly. All statistical tests were two-sided, and *p* < 0.05 was considered statistically significant. The primary analysis was based on complete cases.

### 2.4. Ethical Considerations

This study was approved by the Regional Research Ethics Committee of Qassim Province (registration number: 607-45-12047; approval date: 18 May 2026). Given the retrospective, record-based nature of the study, the requirement for individual informed consent was waived by the Ethics Committee. Patient confidentiality was maintained throughout the study, and study data were handled and stored securely.

### 2.5. Reporting Guideline

This study was reported in accordance with the Strengthening the Reporting of Observational Studies in Epidemiology (STROBE) statement for cross-sectional studies.

## 3. Results

A total of 625 patients with T2DM were included in the final analysis. The mean age was 65.72 ± 11.42 years, and 334 (53.4%) were male. Most patients had known HTN (494, 79.0%), uncontrolled glycemic status (485, 77.6%), and uncontrolled/elevated BP (470, 75.2%). The mean documented diabetes duration was 20.31 ± 7.65 years, with a median of 20 years (IQR, 16–27) and a range of 1–40 years. Obesity was present in 232 patients (37.1%). LVH was identified in 146 of 625 patients, corresponding to a prevalence of 23.4% (95% CI, 20.0–26.7%) within this clinically selected echocardiography cohort (Table 1).

Patients with LVH had a longer mean diabetes duration than those without LVH (25.10 ± 7.09 vs. 18.86 ± 7.22 years; mean difference, 6.24 years; 95% CI, 4.91–7.57; *p* < 0.001). Mean SBP was also higher among patients with LVH (137.34 ± 13.82 vs. 132.10 ± 18.38 mmHg; mean difference, 5.23 mmHg; 95% CI, 2.44–8.02; *p* < 0.001), as was mean DBP (83.60 ± 10.52 vs. 74.68 ± 9.51 mmHg; mean difference, 8.92 mmHg; 95% CI, 7.00–10.83; *p* < 0.001). There was no evidence of a difference in mean age, BMI, or HbA1c between patients with and without LVH (Table 2).

In univariable analyses, LVH was more prevalent among males compared with females (28.1% vs. 17.9%, *p* = 0.002), and among patients with uncontrolled/elevated BP compared with those with controlled/normal BP (26.6% vs. 13.5%, *p* < 0.001). Glycemic control, known HTN, and BMI category were not significantly associated with LVH (Table 3).

**Figure 2:**
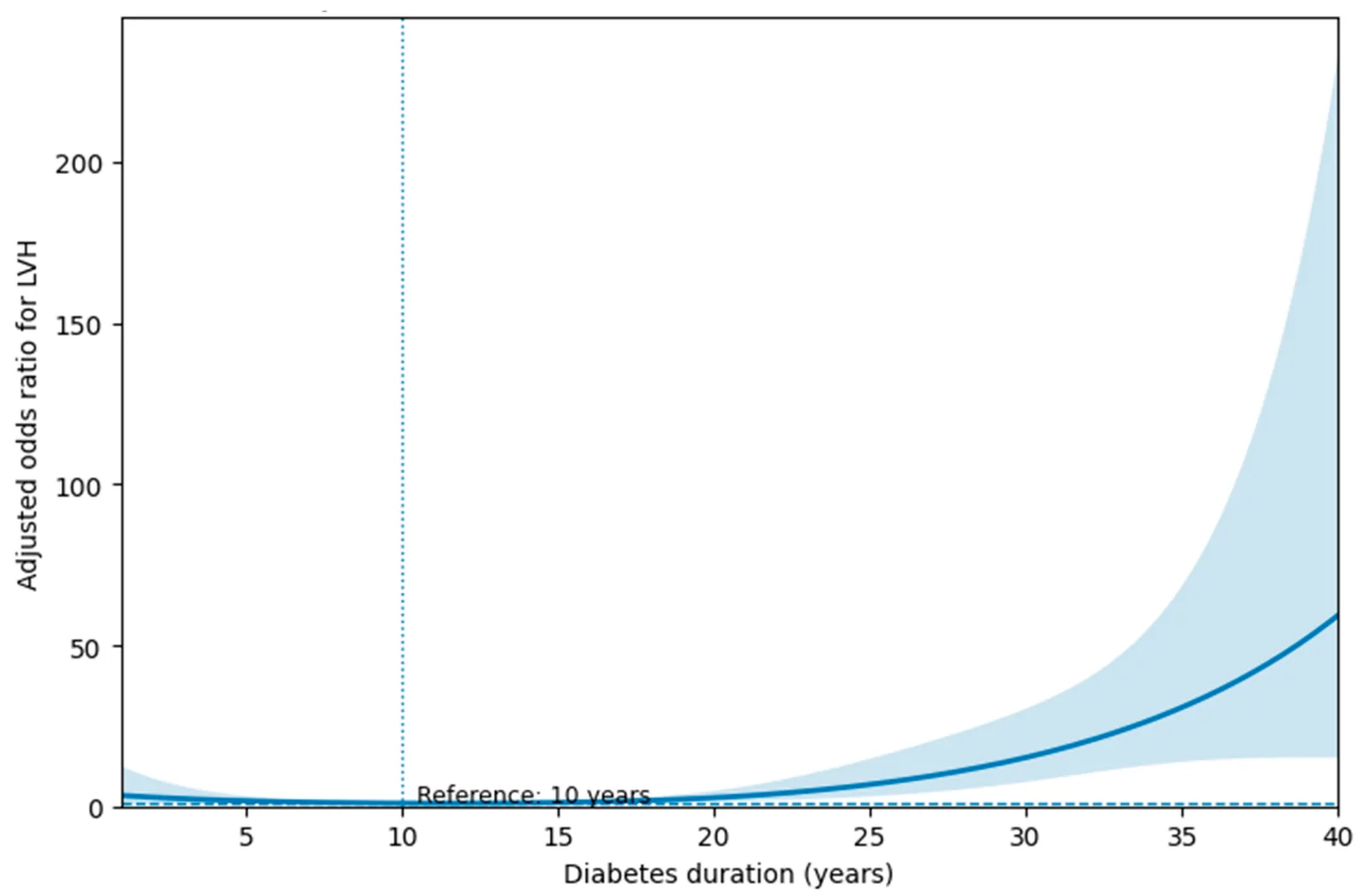
Adjusted association between diabetes duration and LVH. The solid line represents the adjusted odds ratio for LVH across the observed range of diabetes duration, modeled using a natural cubic spline with 4 degrees of freedom, with 10 years as the reference value (OR = 1.0). The shaded area represents the 95% confidence interval. Estimates were adjusted for age, sex, obesity, known hypertension, contemporaneous blood pressure status, and glycemic control.

In the revised multivariable logistic regression model, age was analyzed continuously, whereas diabetes duration was modeled using a natural cubic spline with 4 degrees of freedom because its association with LVH was nonlinear (*p* for nonlinearity = 0.001). Diabetes duration remained significantly associated with LVH overall (*p* < 0.001). The adjusted spline showed that the odds of LVH decreased during the earlier years of diabetes, reached their lowest level at approximately 10 years, and subsequently increased, with a progressively steeper rise at longer diabetes durations (Figure 2). Confidence intervals widened toward the extremes of the observed duration range. Because of this nonlinear pattern, a single odds ratio per one-year increase was not reported.

After adjustment for the other covariates, male sex was associated with higher odds of LVH (aOR = 1.71, 95% CI, 1.11–2.63; *p* = 0.015), and uncontrolled/elevated BP was associated with higher odds of LVH compared with controlled/normal BP (aOR = 2.18, 95% CI, 1.26–3.80; *p* = 0.006). Age, obesity, known HTN, and uncontrolled glycemic status were not independently associated with LVH (Table 4).

The final model included all 625 patients and 146 LVH events. Model discrimination was acceptable, with a C-statistic of 0.780. The Hosmer–Lemeshow goodness-of-fit test showed no evidence of lack of fit (χ^2^ = 13.22, 8 df; *p* = 0.104). No problematic multicollinearity was identified (VIF range, 1.02–1.09).

Sensitivity analyses supported the robustness of the primary findings (Supplementary Table S1). After exclusion of known hypertension from the primary model, the association between uncontrolled/elevated BP and LVH remained materially similar (aOR = 2.32, 95% CI, 1.34–4.01; *p* = 0.003). In models replacing binary BP status with continuous measures, both SBP and DBP were associated with LVH when evaluated separately. When SBP and DBP were entered jointly, DBP remained associated with LVH, whereas SBP did not. Pulse pressure showed an inverse association with LVH in a separate sensitivity model. No evidence of interaction was observed for sex × BP status (*p* = 0.656) or diabetes duration × glycemic control (*p* = 0.515).

## 4. Discussion

In this study, LVH was identified in 23.4% of patients with T2DM without previously documented cardiac disease who underwent clinically indicated echocardiography at a specialized cardiac center. After multivariable adjustment, male sex and uncontrolled/elevated BP were associated with LVH, while diabetes duration showed a significant nonlinear association with LVH. These findings indicate that LVH was common within this clinically selected echocardiography cohort and should be interpreted in the context of its referral-based clinical setting.

The prevalence of LVH observed in our clinically selected cohort is broadly comparable to rates reported in previous studies of diabetes populations. Parsa et al. reported a prevalence of 20.7% among patients with T2DM, Mani et al. reported 25.6% among patients with T2DM, and Muddu et al. reported 19.3% in a cohort of patients with newly diagnosed diabetes that included predominantly T2DM [8,9,16]. A recent study from the Jazan region of Saudi Arabia reported LVH in 30.4% of adults with T2DM undergoing echocardiographic evaluation [11]. This provides a relevant Saudi comparison and further indicates that LVH is common among clinically evaluated patients with T2DM. However, direct comparisons across studies should be made cautiously because of differences in referral setting, age distribution, HTN burden, inclusion and exclusion criteria, and methods used to assess and define LVH. Notably, Parsa et al. assessed LVH using electrocardiographic criteria, whereas the present study used echocardiographic assessment [8]. These clinical and methodological differences may partly account for variation in the reported prevalence estimates.

Diabetes duration was significantly associated with LVH. Patients with LVH had a longer mean diabetes duration than those without (25.10 ± 7.09 vs. 18.86 ± 7.22 years; *p* < 0.001). In the multivariable analysis, diabetes duration showed a significant nonlinear association with LVH (overall *p* < 0.001; *p* for nonlinearity = 0.001). The adjusted spline indicated that the odds of LVH were lowest at approximately 10 years of diabetes duration and increased progressively thereafter, with a steeper increase at longer durations. This pattern should not be interpreted as establishing a specific diabetes-duration threshold, particularly given the wider confidence intervals at the extremes of the observed duration range. This finding is consistent with previous reports linking longer diabetes duration with adverse cardiac structural changes [8,9]. Previous echocardiographic evidence has also shown that left ventricular structural and functional alterations become more pronounced with increasing T2DM duration [17]. In the present study, however, diabetes duration should be interpreted as a marker of cumulative disease exposure rather than as evidence of a direct causal effect. Because of the cross-sectional design, temporality cannot be established, and the observed association may also reflect cumulative exposure to other metabolic and cardiovascular factors over the course of diabetes. Other studies have demonstrated adverse effects of T2DM on left ventricular structure and cardiovascular function, including alterations in myocardial deformation among patients with coexisting HTN [18,19].

Male sex remained significantly associated with LVH after multivariable adjustment in the present study. Previous studies have demonstrated sex-specific differences in left ventricular remodeling; however, the direction and magnitude of these differences have not been consistent across populations. In a recent study of individuals at high cardiovascular risk, women had a higher prevalence of LVH than men when left ventricular mass was indexed to body surface area, although the observed sex difference varied according to the method used to index left ventricular mass [20]. Differences in population characteristics, cardiovascular risk profiles, and methods used to assess and index LVH may partly account for these contrasting findings. The mechanisms underlying the association observed in the present study cannot be determined from our cross-sectional data and warrant further investigation.

A strong association was observed between uncontrolled/elevated BP and LVH after multivariable adjustment. Patients with uncontrolled/elevated BP had more than twice the adjusted odds of having LVH compared with those with controlled/normal BP, which is consistent with the established relationship between pressure overload and LVH [21,22]. Sustained elevation in BP can increase left ventricular afterload and myocardial wall stress, promoting adaptive myocardial hypertrophy and progressive ventricular remodeling [21,22,23]. However, in the present cross-sectional study, BP was assessed contemporaneously with echocardiography; therefore, temporality cannot be established, and the observed association should not be interpreted as demonstrating that the recorded BP preceded or caused LVH. In addition, residual confounding by antihypertensive treatment and longer-term BP burden cannot be excluded.

Notably, uncontrolled/elevated BP remained significantly associated with LVH after multivariable adjustment, whereas known HTN history was not independently associated with LVH in the same model. These variables represent different aspects of BP exposure: known HTN reflects a documented clinical history, whereas the BP category used in this study reflects measurements obtained within the same calendar month as the echocardiographic examination. Therefore, these findings should not be interpreted as indicating that current BP status is more important than a history of HTN. HTN remains an important contributor to cardiovascular risk among individuals with diabetes, and appropriate BP management is a key component of cardiovascular risk reduction [24,25]. Because information on HTN duration, severity, cumulative BP exposure, and antihypertensive treatment was not consistently available, the independent associations of these measures should be interpreted cautiously.

Despite the high prevalence of uncontrolled glycemic status, glycemic control was not significantly associated with LVH after multivariable adjustment. This null finding should be interpreted cautiously because glycemic status in the primary analysis was based on HbA1c measured within the same calendar month as echocardiography and was dichotomized at 7.0%. In addition, 77.6% of the cohort was classified as having uncontrolled glycemic status, which may have limited discrimination between groups, and data on cumulative or long-term glycemic exposure were not available. In a sensitivity analysis treating HbA1c as a continuous variable, the association remained nonsignificant (aOR per 1% increase = 1.01, 95% CI: 0.91–1.13; *p* = 0.799). Therefore, the absence of a significant association should not be interpreted as evidence that long-term glycemic exposure is unrelated to cardiac remodeling. A recent scoping review from Pakistan also documented substantial variability in glycemic control, awareness, and treatment adherence among patients with T2DM [26]. Chronic hyperglycemia may contribute to cardiovascular structural changes through multiple indirect mechanisms, including endothelial dysfunction, vascular remodeling, and alterations in arterial and cardiac function [27,28,29].

Obesity (BMI ≥ 30 kg/m^2^) was also not significantly associated with LVH in the present study. Although obesity may increase cardiac workload and contribute to adverse cardiac remodeling, its relationship with LVH is complex and may be influenced by coexisting hemodynamic and metabolic factors. In addition, BMI does not directly characterize body fat distribution or visceral adiposity. These potential explanations were not evaluated in the present study, and the absence of a statistically significant association should therefore be interpreted cautiously.

This study has several strengths. First, the study included all eligible patients identified during the study period, providing a substantial clinical sample for evaluating LVH and its associated factors. Second, the study was conducted at a specialized cardiac center serving the Qassim region of Saudi Arabia. Third, LVH was assessed using echocardiography interpreted by cardiologists in accordance with established ASE/EACVI recommendations. Finally, the study evaluated multiple demographic and clinical factors, and multivariable logistic regression allowed assessment of their associations with LVH while adjusting for other variables included in the model.

Nevertheless, this study has several limitations. First, the cross-sectional design precludes establishing temporal or causal relationships between the factors examined and LVH. Second, the retrospective nature of data collection may have introduced documentation bias and limited the availability and completeness of some clinical variables. Of the 878 patients with T2DM and no documented cardiac disease assessed for eligibility, 253 (28.8%) were excluded because one or more required variables were missing or unavailable. This complete-case approach may have introduced selection bias if excluded patients differed systematically from those included. Because individual-level data for the excluded patients were not retained in the final analytical dataset, these differences could not be evaluated and the direction of any resulting bias cannot be determined.

Third, the study included patients with T2DM who underwent echocardiography at a specialized cardiac center based on clinical indications rather than through routine echocardiographic screening. This may have introduced selection and referral bias because patients referred for echocardiography may differ in cardiovascular risk from the broader population of individuals with T2DM. Such referral could result in overrepresentation of patients with clinical characteristics, prompting echocardiographic evaluation; however, without a non-referred comparison group, the magnitude and direction of its effect on the observed LVH prevalence cannot be determined. Consequently, the observed prevalence of LVH should not be interpreted as a population-based prevalence estimate for all individuals with T2DM in Saudi Arabia, and the generalizability of the findings may be limited.

Fourth, echocardiographic measurements were obtained from the final cardiologist-interpreted clinical reports, and no study-specific re-reading or formal assessment of interobserver variability was performed. Therefore, measurement variability inherent in routine clinical echocardiographic interpretation cannot be excluded. In addition, LV mass was indexed to body surface area according to the ASE/EACVI approach used in the study, and alternative indexation methods, such as height-based indexation, could not be evaluated, because continuous LV mass and LVMI values were not retained in the analytical dataset.

Fifth, information on pharmacological treatment was not consistently or completely documented in the available medical records. Therefore, potentially relevant glucose-lowering and cardiovascular therapies, including sodium-glucose co-transporter 2 inhibitors, angiotensin-converting enzyme inhibitors/angiotensin II receptor blockers, mineralocorticoid receptor antagonists, and other antihypertensive therapies, could not be reliably evaluated or included in the adjusted analysis. In addition, renal function, albuminuria, lipid profile, smoking status, anemia, and the duration and severity of HTN were not sufficiently available for inclusion in the analysis. BP status was based on measurements obtained within the same calendar month as echocardiography and therefore represents contemporaneous BP rather than long-term or cumulative BP burden, limiting temporal interpretation of its association with LVH. Consequently, residual confounding by pharmacological treatment and these unmeasured or incompletely measured clinical factors cannot be excluded, and the direction and magnitude of this residual confounding cannot be determined from the available data.

Finally, the study did not include a non-diabetic comparison group. Therefore, the findings characterize LVH and its associated factors within this clinically selected T2DM cohort but do not allow estimation of the excess prevalence of LVH attributable to T2DM relative to individuals without diabetes.

From a clinical perspective, the 23.4% prevalence of LVH indicates that LVH was frequently identified among patients with T2DM who had already been selected for clinically indicated echocardiography. This finding should not be interpreted as supporting routine echocardiographic screening of all patients with T2DM. The associations observed with uncontrolled/elevated BP and longer diabetes duration may help identify clinical characteristics, warranting greater attention to cardiovascular risk; however, the cross-sectional design and clinically selected sample do not establish which patients should undergo echocardiographic screening or a specific diabetes-duration threshold at which echocardiography should be performed. Prospective studies in broader, unselected T2DM populations are needed to define whether targeted echocardiographic screening is beneficial and, if so, to establish appropriate selection criteria and timing.

## 5. Conclusions

LVH was common among this clinically selected group of patients with T2DM undergoing clinically indicated echocardiography. After multivariable adjustment, male sex and uncontrolled/elevated BP were associated with LVH, while diabetes duration showed a significant nonlinear association with LVH. Although causal relationships cannot be established from this cross-sectional study, the observed association between uncontrolled/elevated BP and LVH reinforces the clinical importance of appropriate blood pressure management as part of comprehensive cardiovascular risk reduction in patients with T2DM.

## Figures and Tables

**Table 1:** Demographic and clinical characteristics of the study population (*n* = 625).

Variables		Value
Age, years, mean ± SD		65.72 ± 11.42
Diabetes duration, years, mean ± SD		20.31 ± 7.65
Diabetes duration, years, median (IQR)		20 (16–27)
Diabetes duration, range		1–40
Sex, *n* (%)	Male	334 (53.4)
	Female	291 (46.6)
Known HTN, *n* (%)	No	131 (21.0)
	Yes	494 (79.0)
Glycemic control (HbA1c), *n* (%)	Controlled	140 (22.4)
	Uncontrolled	485 (77.6)
BP status, *n* (%)	Controlled/normal	155 (24.8)
	Uncontrolled/elevated	470 (75.2)
BMI category, *n* (%)	Underweight	12 (1.9)
	Normal	201 (32.2)
	Pre-obesity	180 (28.8)
	Obese	232 (37.1)
LVH status, *n* (%)	Yes	146 (23.4)
	No	479 (76.6)

Controlled/normal BP was defined as SBP < 130 mmHg and DBP < 80 mmHg; uncontrolled/elevated BP was defined as SBP ≥ 130 mmHg or DBP ≥ 80 mmHg. Data are presented as mean ± SD, median (IQR), or *n* (%), as appropriate.

**Table 2:** Comparison of continuous clinical characteristics according to LVH status.

Variable	LVH (*n* = 146)	No LVH (*n* = 479)	Mean Difference (95% CI) ^	*p* Value
Age, years	66.56 ± 10.68	65.47 ± 11.64	1.09 (−0.94 to 3.12)	0.291
SBP, mmHg	137.34 ± 13.82	132.10 ± 18.38	5.23 (2.44 to 8.02)	<0.001 *
DBP, mmHg	83.60 ± 10.52	74.68 ± 9.51	8.92 (7.00 to 10.83)	<0.001 *
BMI, kg/m^2^	27.82 ± 5.64	28.75 ± 7.30	−0.93 (−2.06 to 0.20)	0.107
HbA1c, %	8.17 ± 1.97	8.07 ± 1.78	0.10 (−0.26 to 0.46)	0.581
Diabetes duration, years	25.10 ± 7.09	18.86 ± 7.22	6.24 (4.91 to 7.57)	<0.001 *

Data are presented as mean ± standard deviation. ^ Mean differences were calculated as LVH minus no LVH. Variance homogeneity was assessed, and Welch’s independent-samples *t*-test was used to accommodate unequal variances where applicable. All tests were two-sided. * *p* values < 0.05 were considered statistically significant.

**Table 3:** Univariable associations between demographic and clinical characteristics and LVH.

Variable	Category	LVH, *n* (%)	No LVH, *n* (%)	Total, *n*	*p* Value
Sex	Male	94 (28.1)	240 (71.9)	334	0.002 *
	Female	52 (17.9)	239 (82.1)	291	
Glycemic control (HbA1c)	Controlled	31 (22.1)	109 (77.9)	140	0.699
	Uncontrolled	115 (23.7)	370 (76.3)	485	
Known HTN	Yes	120 (24.3)	374 (75.7)	494	0.285
	No	26 (19.8)	105 (80.2)	131	
BP status	Controlled/normal	21 (13.5)	134 (86.5)	155	<0.001 *
	Uncontrolled/elevated	125 (26.6)	345 (73.4)	470	
BMI category	Underweight	3 (25.0)	9 (75.0)	12	0.416 ^
	Normal	50 (24.9)	151 (75.1)	201	
	Pre-obesity	47 (26.1)	133 (73.9)	180	
	Obese	46 (19.8)	186 (80.2)	232	

Controlled/normal BP was defined as SBP < 130 mmHg and DBP < 80 mmHg; uncontrolled/elevated BP was defined as SBP ≥ 130 mmHg or DBP ≥ 80 mmHg. *p* values were obtained using Pearson’s chi-square test unless otherwise indicated. ^ Fisher’s exact test was used for the four-category BMI comparison because of the small underweight group. * *p* values < 0.05 were considered statistically significant.

**Table 4:** Univariable and multivariable logistic regression analyses of factors associated with LVH (*n* = 625; LVH events = 146).

Variable	Unadjusted OR (95% CI)	*p* Value	Adjusted OR (95% CI)	*p* Value
Age, per year	1.01 (0.99–1.03)	0.312	0.99 (0.97–1.01)	0.217
Male sex	1.80 (1.23–2.64)	0.003 *	1.71 (1.11–2.63)	0.015 *
Obesity (BMI ≥ 30.0 kg/m^2^)	0.72 (0.49–1.08)	0.110	0.68 (0.44–1.07)	0.093
Known HTN	1.30 (0.80–2.09)	0.286	1.41 (0.81–2.45)	0.224
Diabetes duration, natural cubic spline	-	<0.001 ^*	-	<0.001 ^*
Uncontrolled/elevated BP	2.31 (1.40–3.82)	0.001 *	2.18 (1.26–3.80)	0.006 *
Uncontrolled HbA1c	1.09 (0.70–1.71)	0.699	1.00 (0.60–1.67)	0.999

Controlled/normal BP was defined as SBP < 130 mmHg and DBP < 80 mmHg; uncontrolled/elevated BP was defined as SBP ≥ 130 mmHg or DBP ≥ 80 mmHg. * *p* values < 0.05 were considered statistically significant. Reference categories: female sex; non-obesity (BMI < 30 kg/m^2^); no known HTN; controlled/normal BP; and controlled HbA1c. Age was modeled continuously per 1-year increase. Diabetes duration was modeled using a natural cubic spline with 4 degrees of freedom because of evidence of nonlinearity; therefore, a single OR is not reported. ^ *p* values for diabetes duration represent the overall association; in the adjusted model, *p* for nonlinearity = 0.001. The multivariable model included 625 patients with 146 LVH events. Model C-statistic = 0.780. Hosmer–Lemeshow goodness-of-fit test: χ^2^ = 13.22, df = 8, *p* = 0.104.

## Data Availability

The original contributions presented in this study are included in the article. Further inquiries can be directed to the corresponding author.

## Supplementary Materials

The following supporting information can be downloaded at https://www.mdpi.com/article/10.3390/biomedicines14102308/s1. Table S1: Sensitivity and interaction analyses for factors associated with left ventricular hypertrophy.

## Abbreviations

The following abbreviations are used in this manuscript:

T2DM	Type 2 diabetes mellitus
LVH	Left ventricular hypertrophy
HTN	Hypertension
BP	Blood pressure
SBP	Systolic blood pressure
DBP	Diastolic blood pressure
HbA1c	Hemoglobin A1c
BMI	Body mass index
LVMI	Left ventricular mass index
aORs	Adjusted odds ratios
CIs	Confidence intervals
SPSS	Statistical Package for the Social Sciences
SDs	Standard deviations
ASE/EACVI	American Society of Echocardiography and the European Association of Cardio-vascular Imaging
