# Supplementary material for: Echocardiographic Left Ventricular Hypertrophy Among Patients with Type 2 Diabetes Undergoing Clinically Indicated Echocardiography at a Saudi Cardiac Center: Prevalence and Associated Factors
Source: Biomedicines. 2026 Oct 11;14(10):2308. doi: 10.3390/biomedicines14102308 (PMC13702396; doi:10.3390/biomedicines14102308)
Supplement: Supplementary file 1 [file biomedicines-14-02308-s001.zip › biomedicines-4567087-supplementary.pdf]

Supplementary Table S1. Sensitivity and interaction analyses for factors associated with left ventricular hypertrophy

**Analysis**	**Exposure / comparison**	**Adjusted OR (95% CI)**	***p* Value**
Primary model	Uncontrolled/elevated BP vs. controlled/normal BP	2.18 (1.26–3.80)	0.006
Model excluding history of hypertension	Uncontrolled/elevated BP vs. controlled/normal BP	2.32 (1.34–4.01)	0.003
Continuous BP model	SBP, per 10-mmHg increase	1.16 (1.03–1.31)	0.013
Continuous BP model	DBP, per 10-mmHg increase	2.73 (2.12–3.51)	<0.001
Pulse-pressure model	Pulse pressure, per 10-mmHg increase	0.83 (0.73–0.95)	0.006
Joint SBP–DBP model	SBP, per 10-mmHg increase	0.95 (0.82–1.10)	0.511
Joint SBP–DBP model	DBP, per 10-mmHg increase	2.81 (2.14–3.69)	<0.001
Interaction analysis	Sex × BP status	—	0.656 *
Interaction analysis	Diabetes duration × glycemic control	—	0.515 *
Continuous HbA1c sensitivity analysis	HbA1c, per 1% increase	1.01 (0.91–1.13)	0.799

OR, odds ratio; CI, confidence interval; BP, blood pressure; SBP, systolic blood pressure; DBP, diastolic blood pressure. All sensitivity models were adjusted for the prespecified covariates in the primary multivariable model, with modification of the BP or hypertension-history term as indicated. Age was modeled continuously, and diabetes duration was modeled using a natural cubic spline with 4 degrees of freedom. In the separate continuous BP models, SBP, DBP, and pulse pressure were entered individually in place of the binary BP-status variable. In the joint SBP–DBP model, continuous SBP and DBP were entered simultaneously. ORs for continuous BP measures are reported per 10-mmHg increase. The OR for continuous HbA1c is reported per 1% increase. * *p* values for interaction were obtained from likelihood-ratio tests comparing models with and without the corresponding interaction terms. Interaction terms were not retained in the primary model because there was no evidence of effect modification.
